# Impaired topology and connectivity of grey matter structural networks in major depressive disorder: evidence from a multi-site neuroimaging data-set

**DOI:** 10.1192/bjp.2024.41

**Published:** 2024-05

**Authors:** Jing-Yi Long, Kun Qin, Nanfang Pan, Wen-Liang Fan, Yi Li

**Affiliations:** Wuhan Mental Health Center, Wuhan, China; Affiliated Wuhan Mental Health Center, Tongji Medical College of Huazhong University of Science and Technology, Wuhan, China; and Research Center for Psychological and Health Sciences, China University of Geosciences, Wuhan, China; Department of Radiology, Taihe Hospital, Hubei University of Medicine, Shiyan, China; Huaxi Magnetic Resonance Research Center (HMRRC), West China Hospital of Sichuan University, Chengdu, China; Department of Radiology, Union Hospital, Tongji Medical College, Huazhong University of Science and Technology, Wuhan, China; and Department of Radiology, Hubei Province Key Laboratory of Molecular Imaging, Wuhan, China

**Keywords:** Magnetic resonance imaging, graph theory, depression, machine learning, brain network

## Abstract

**Background:**

Major depressive disorder (MDD) has been increasingly understood as a disruption of brain connectome. Investigating grey matter structural networks with a large sample size can provide valuable insights into the structural basis of network-level neuropathological underpinnings of MDD.

**Aims:**

Using a multisite MRI data-set including nearly 2000 individuals, this study aimed to identify robust topology and connectivity abnormalities of grey matter structural network linked to MDD and relevant clinical phenotypes.

**Method:**

A total of 955 MDD patients and 1009 healthy controls were included from 23 sites. Individualised structural covariance networks (SCN) were established based on grey matter volume maps. Following data harmonisation, network topological metrics and focal connectivity were examined for group-level comparisons, individual-level classification performance and association with clinical ratings. Various validation strategies were applied to confirm the reliability of findings.

**Results:**

Compared with healthy controls, MDD individuals exhibited increased global efficiency, abnormal regional centralities (i.e. thalamus, precentral gyrus, middle cingulate cortex and default mode network) and altered circuit connectivity (i.e. ventral attention network and frontoparietal network). First-episode drug-naive and recurrent patients exhibited different patterns of deficits in network topology and connectivity. In addition, the individual-level classification of topological metrics outperforms that of structural connectivity. The thalamus-insula connectivity was positively associated with the severity of depressive symptoms.

**Conclusions:**

Based on this high-powered data-set, we identified reliable patterns of impaired topology and connectivity of individualised SCN in MDD and relevant subtypes, which adds to the current understanding of neuropathology of MDD and might guide future development of diagnostic and therapeutic markers.

Major depressive disorder (MDD) is a common and debilitating mental illness that affects over 320 million people worldwide.^1^ The main clinical characteristics of MDD include depressed mood, guilt, worthlessness and anhedonia, accounting for profound social, interpersonal and occupational impacts. However, the neuropathological mechanisms of MDD remain unclear. Although a number of neuroimaging studies have demonstrated that MDD is underpinned by brain structural and functional alterations associated with emotional and cognitive impairments,^2,3^ the limited sample size has largely impeded the identification of reliable biomarkers in such a highly heterogeneous population. At least thousands of individuals are necessary for a high-quality and reproducible neuroimaging study to identify differences between cohorts of participants.^4^

Multisite consortiums have provided a unique opportunity to explore robust and generalisable multimodal neuroimaging biomarkers in various psychiatric disorders with sufficient statistical power and cross-site validations. For example, based on data from 20 sites, the Enhancing NeuroImaging Genetics through Meta-Analysis (ENIGMA) consortium identified a reliable pattern of brain structural alterations in the frontal, temporal, limbic and visual regions in MDD.^5^ The Disease Imaging Data Archiving – Major Depressive Disorder (DIDA-MDD) working group detected reproducible brain functional abnormalities in the visual, orbitofrontal and sensorimotor cortices using multisite resting-state functional MRI scans from 1434 individuals.^6^ Widespread white matter microstructural impairments underlying structural disconnectivity in MDD were thoroughly examined through a coordinated multisite diffusion tensor imaging study,^7^ also suggesting potential concurrent alterations in grey matter across the brain.^8^ These high-quality multisite neuroimaging studies provided valuable insights into the neural mechanisms of MDD, facilitating the identification of robust illness biomarkers and potential treatment targets.

In the past decade, the pathophysiology of MDD has been increasingly understood as a disruption of brain networks instead of independent regional abnormalities.^9^ Complex brain networks are typically represented by interregional structural or functional connection matrices, from which network topological organisation and focal connectivity patterns can be investigated. In addition to functional or white matter networks that have been widely studied in MDD, growing evidence supported that depression is associated with grey matter structural networks (i.e. structural covariance network [SCN]) that measure covariation in brain morphology between regions. For example, Chen et al found that first-episode MDD patients exhibited topological alterations of SCN in the superior frontal gyrus and paracentral lobule, compared to controls.^10^ Xiong et al demonstrated that impaired small-world architecture of SCN could serve as a trait imaging marker of MDD.^11^

However, most previous studies estimated structural covariance by calculating interregional morphological correlations across a group of individuals, which can only generate one SCN for a given group. Group-level SCN neglects the heterogeneity of MDD and cannot investigate the brain–behaviour relationship or make individual-level classification. Moreover, examining focal connectivity patterns is largely limited in most group-level SCN studies, because the connection of group SCN represents inter-individual morphological similarity which lacks biological meanings. Thus, establishing individualised SCN (iSCN) can be more informative given its role in personalised diagnosis and treatment. Although there have been increasing numbers of works examining iSCN in MDD patients,^12,13^ the sample size and statistical power were limited.

In the present study, we aimed to identify robust abnormalities of iSCN in MDD using a multisite structural MRI data-set including nearly 2000 participants. Network topological metrics, as well as focal connectivity, were harmonised and compared between patients and controls. Clinical subgroups including first-episode drug-naive (FEDN) and recurrent patients were separately investigated. Given the existence of multiple controversial alternatives in various stages of the iSCN analytic pipeline, particularly in brain parcellation, brain mask application, image spatial smoothing and covariate selection, we conducted a reproducibility test to assess whether our findings were influenced by these methodological choices. Machine learning analysis implemented through support vector machine (SVM) was employed to demonstrate the diagnostic value of network topology and connectivity. The choice of SVM is rooted in its effectiveness in high-dimensional spaces, robustness to overfitting, global optimality, balanced handling of bias and variance and, notably, its widespread popularity in the neuroimaging field.^14^ The close association between deficits in cognitive and affective processing patterns and depressive-like behaviours in MDD has been well established.^15^ Previous studies have consistently supported the link between brain network abnormalities and cognitive-emotional dysfunction.^16^ Consequently, our hypothesis posits that individuals with MDD would exhibit alterations in network topology and connectivity of regions implicated in emotion processing and cognitive function. Different patterns of iSCN abnormalities might be observed between FEDN and recurrent patients.

## Materials and methods

### Participants

The data-set utilised in this study was sourced from the REST-meta-MDD consortium, encompassing MRI scans from 25 hospitals nationwide. Following meticulous data cleaning and selection (Supplementary Methods I available at https://doi.org/10.1192/bjp.2024.41), our analysis ultimately comprised a subsample of 955 MDD patients and 1009 healthy controls from 23 sites. Data collection of all study cohorts was approved by their local Institutional Review Boards (IRBs). The diagnoses of MDD were confirmed by experienced psychiatrists at each local site using the Structured Clinical Interview for DSM-IV disorders (SCID-IV). All included participants aged 18–65 years old and had a 17-item Hamilton Depression Rating Scale (HAMD-17) total score ≥18 at the time of screening. Among all the MDD patients included, 263 were first-episode individuals, 201 were recurrent individuals, 374 people were medication-naive and 261 people were treated. Demographic and clinical characteristics are shown in Table 1 and Supplementary Figure 1.

**Table 1:** Demographic and clinical characteristics of the included sample

Variable	MDD	Healthy controls	*P*-value
Sample size (*N*)	955	1009	−
Age (year, mean ± s.d.)	34.6 ± 11.7	34.1 ± 13.7	0.510
Gender (*N* female, %)	604 (63.2%)	607 (60.2%)	0.160
HAMD (mean ± s.d.)	23.8 ± 4.6	−	−
Duration of illness (month, mean ± s.d.)	32.7 ± 54.4	−	−
Episode status (*N*, %)	−	−	−
First-episode	426 (44.6%)	−	−
Recurrent	201 (21.1%)	−	−
Unknown	328 (34.3%)	−	−
Medication status (*N*, %)	−	−	−
Medication-naive	374 (39.2%)	−	−
Treated	261 (27.3%)	−	−
Unknown	320 (33.5%)	−	−

MDD, major depressive disorder; HAMD, Hamilton Depression Rating Scale.

## Ethics

The authors assert that all procedures contributing to this work comply with the ethical standards of the relevant national and institutional committees on human experimentation and with the Helsinki Declaration of 1975 as revised in 2008.

## Consent

Written informed consent was obtained from all participants at each hospital.

### Image acquisition and processing

Three-dimensional high-resolution T1-weighted brain structural images were acquired at each site. Scanner and image acquisition parameters are presented in Supplementary Table 1. The REST-meta-MDD consortium performed image preprocessing based on a unified pipeline using the DPABI toolbox (http://rfmri.org/DPABI). The raw structural images underwent initial correction for field inhomogeneities using the Non-parametric Non-uniform Intensity Normalisation (N3) algorithm.^17^ This algorithm employs an iterative approach to estimate and eliminate both the multiplicative bias field and the distribution of true tissue intensities. Subsequently, the images were automatically segmented into grey matter, white matter and cerebrospinal fluid via an iterative mixture model cluster analysis embedded in the SPM software, on the basis of both prior tissue probability and signal intensity information.^18^ The segmented grey matter maps in native space were spatially normalised to Montreal Neurological Institution (MNI) space using diffeomorphic anatomical registration through exponentiated lie algebra (DARTEL) non-linear registration followed by modulation.^19^ The modulated grey matter maps were further smoothed using a Gaussian kernel of 8-mm full width at half maximum (FWHM).

### Individualised structural covariance network (iSCN) construction

Brain complex networks consist of nodes and edges. In our study, the nodes of the iSCN were defined as 246 cortical and subcortical regions based on the parcellation of the Brainnetome atlas. The inter-regional structural covariance (i.e. structural connectivity) represented edges, calculated as the similarities of grey matter volume (GMV) probability distribution between each pair of regions. Specifically, kernel density estimation (KDE) was first employed to estimate the probability density function (PDF) to reflect the GMV distribution in each region. In this process, the Gaussian kernel was used by default, and a conservative number of sampling points was set to 512 as suggested by previous studies.^20,21^ Next, we measured the differences between pairwise PDFs of regional GMV via the symmetric Kullback–Leibler divergence (KLD). The interregional KLD values were subsequently transformed to Kullback–Leibler similarity (KLS) values which ranged from 0 to 1 (KLS = e^−KLD^). Finally, a symmetric 246 × 246 similarity matrix was established to represent the iSCN for each participant.

### Analysis of topological profiles

Global and nodal topological metrics of each iSCN were calculated using the Brain Connectivity Toolbox (https://github.com/brainlife/BCT). Global topological metrics included global efficiency, clustering coefficient, small-worldness, modularity and assortativity, which reflect five domains of the brain network architecture including integration, segregation, small-worldness, modularity and resilience. Nodal topological metrics contained degree, betweenness and eigenvector centrality of each region, reflecting the importance of individual region in the network. A detailed description of the above topological metrics is presented in Supplementary Methods II. To remove weak and possibly spurious edges, iSCN was first thresholded and binarised based on network density. The density-based thresholding strategy retained the K% strongest edges, which could ensure that each participant had the same number of edges and could facilitate subsequent group comparisons. To avoid the bias of single threshold selection, we applied a range of dynamic density thresholds (K = 12–39 with an interval of 1) which ensured both network connectedness and small-worldness. Specifically, the upper and lower bounds of the threshold range were set according to the following criteria: (a) all the nodes were connected to others, and (b) the small-worldness of all the networks was greater than 1.1 (Supplementary Figure 2). The area under curve (AUC) value of each topological metric over dynamic density thresholds was calculated for subsequent statistical analysis. Given the significant bias derived from the site effects in multisite neuroimaging data, ComBat harmonisation was applied (Supplementary Methods III). A general linear model was used to evaluate case-control differences in harmonised topological metrics. Age and gender were set as covariates. We used the Bonferroni correction (corrected P < 0.05) to control for multiple comparisons.

### Analysis of connectivity patterns

Between-group differences in network structural connectivity were investigated using network-based statistics (NBS) analysis, which could identify connected focal network components with significantly altered connectivity in MDD relative to healthy controls.^22^ Prior to the NBS analysis, ComBat harmonisation was performed on each connectivity to minimise the site effects (Supplementary Methods III). We first compared all connections within the iSCN to gather connections exhibiting significant inter-group differences (two-tailed *P* < 0.001). The breadth first search was subsequently performed to identify any connected subnetwork components within these connections. To further examine the significance level of these connected subnetwork components, non-parametric permutation testing with 10 000 randomisations was performed, and the size of the maximal subnetwork component was recorded for each randomisation. We reported significant components whose sizes rank as the top 5% of the 10 000 maximal subnetwork components derived from permutations (corrected-*P* < 0.05). Nodes within each significant subnetwork component were further grouped into seven canonical intrinsic functional networks based on the Yeo atlas to reveal inter- and intra-network connectivity patterns. The seven intrinsic functional networks comprised default mode network (DMN), ventral attention network (VAN), visual network (VN), dorsal attention network (DAN), somatomotor network (SMN), frontoparietal network (FPN) and limbic network (LN).^23^

### Subgroup analysis

For the subgroup analyses, FEDN and recurrent patients were separately examined. The healthy controls for each subgroup were allocated from the same sites as the patients for a balance of site effect harmonisation. Specifically, 263 FEDN patients versus 532 healthy controls from 10 sites and 201 recurrent patients versus 562 healthy controls from 10 sites were included (Supplementary Tables 2 and 3). We used the identical statistical model and significance level to examine between-group differences in both topological profiles and connectivity patterns of iSCN.

### Individual-level classification

In addition to group-level statistical comparisons, we performed individual-level machine learning analysis to separately examine the diagnostic value of iSCN topology and connectivity. Three classification tasks were determined to distinguish between MDD versus healthy controls, FEDN MDD versus healthy controls and recurrent MDD versus healthy controls, resulting in a total of six models. The machine learning model was set as a linear support vector machine (Supplementary Methods IV). We applied stratified ten-fold cross-validation to split the data-set into training and test sets. Classification performance was examined based on accuracy, sensitivity, specificity and AUC across ten folds. We extracted the mean absolute weight of each feature across ten folds from each trained model and mapped the top ten regions contributing most to the classification tasks. For the models based on topology, the region-level contribution was determined based on the average weights of three regional centralities. For the models based on network connectivity, the region-level contribution was derived from the average weights of all connectivity linked to the region.

### Association with symptom severity

We examined the association between depressive symptom severity (i.e. HAMD-17 scores) and harmonised network measures using partial correlation controlling for age and gender. Given the ample statistical power of our data-set, we extended our association analysis to encompass all metrics, not just limited to those demonstrating significant between-group differences. This decision was driven by our aim to thoroughly explore potential associations between imaging and clinical variables. We separately examined clinical association with all the topological measures and whole-brain structural connectivity values. Bonferroni correction was applied to control for type I error of multiple correlations (corrected-*P* < 0.05).

### Reproducibility test

The reproducibility tests were performed by considering several alternative methodological choices. First, we used the 3rd version of automated anatomical labelling (AAL3) atlas for brain parcellation which contained 140 cerebral regions to examine whether the findings were influenced by different node definition. Second, in order to explore iSCN disruptions in the cerebellum and examine whether the cerebral findings were influenced by introducing cerebellar regions, we used another version of the Brainnetome atlas which contained cerebellar subfields. Third, it is controversial to perform spatial smoothing during image preprocessing for analysis of iSCN. We tested the reproducibility of results using non-smoothed images. Fourth, we included the total intracranial volume (TIV) as an additional covariate for group-level comparisons, as TIV is thought to correlate with the regional brain volume which is the measure we used to construct iSCN.

## Results

### Network topological deficits

#### MDD patients versus healthy controls

**Fig. 1:**
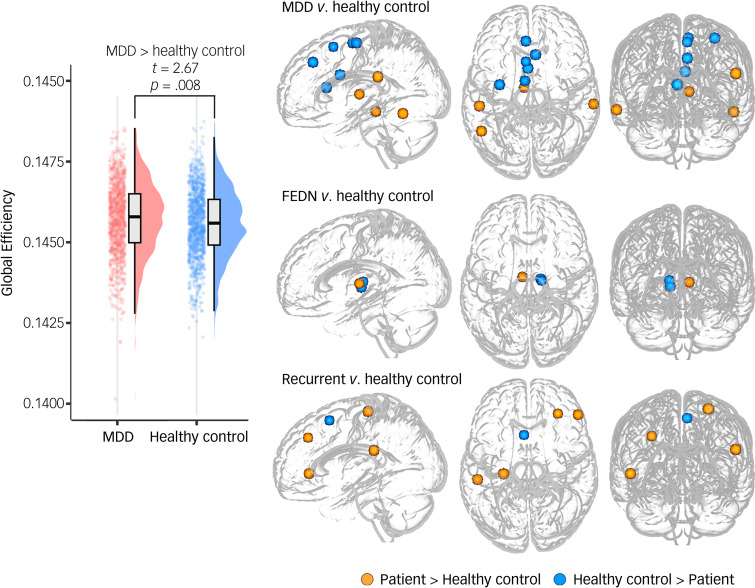
Group differences in global and nodal topological metrics. The left panel shows the significant case-control difference in global efficiency. Nodes with significant differences after Bonferroni correction in either degree, betweenness and eigenvector centrality are presented in the right panel. The colour of the nodes indicates the direction of the group differences. MDD, major depressive disorder; FEDN, first-episode drug naïve.

Increased global efficiency (*P* = 0.008, Cohen's d = 0.12) was identified in MDD patients compared with healthy controls. MDD patients exhibited significantly decreased regional centralities in the left medial superior frontal gyrus (SFG), left precentral gyrus (PCG) and bilateral middle cingulate cortex (MCC), while increased regional centralities were observed in the bilateral lateral temporal cortex (LTC), left inferior parietal lobule (IPL) and left thalamus (Fig. 1 and Table 2).

**Table 2:** Between-group differences in regional topological centralities

Region	Metric	Cohen's *d*	*P*-value (Bonferroni corrected)
***MDD < healthy controls***			
L SFG, medial area 8	Degree centrality	−0.19	0.0062
L SFG, medial area 6	Degree centrality	−0.17	0.0385
L SFG, medial area 9	Degree centrality	−0.18	0.0216
L PrG, caudal dorsolateral area 6	Degree centrality	−0.22	0.0004
	Eigenvector centrality	−0.20	0.0026
L CG, rostroventral area 24	Degree centrality	−0.25	<0.0001
	Eigenvector centrality	−0.26	<0.0001
R CG, rostroventral area 24	Degree centrality	−0.23	<0.0001
	Eigenvector centrality	−0.23	<0.0001
***MDD > healthy controls***			
R MTG, caudal area 21	Degree centrality	0.22	0.0006
	Betweenness centrality	0.19	0.0152
L ITG, extreme lateroventral area 37	Degree centrality	0.20	0.0039
	Eigenvector centrality	0.19	0.0071
L IPL, rostroventral area 40	Degree centrality	0.20	0.0026
L medial prefrontal thalamus	Degree centrality	0.21	0.0002
	Eigenvector centrality	0.17	0.0143
***FEDN < healthy controls***			
R lateral prefrontal thalamus	Degree centrality	−0.33	0.0058
R pre-motor thalamus	Eigenvector centrality	−0.35	0.0008
***FEDN > healthy controls***			
L medial prefrontal thalamus	Betweenness centrality	0.27	0.0284
***Recurrent < healthy controls***			
L SFG, medial area 8	Eigenvector centrality	−0.32	0.0199
***Recurrent > healthy controls***			
L MFG, dorsal area 9/46	Degree centrality	0.33	0.0124
R IFG, rostral area 45	Degree centrality	0.37	0.0011
	Eigenvector centrality	0.33	0.0091
L IPL, rostroventral area 40	Degree centrality	0.34	0.0059
L PrG, upper limb area 4	Eigenvector centrality	0.32	0.0263

MDD, major depressive disorder; FEDN, first-episode drug-naive; L, left; R, right; SFG, superior frontal gyrus; MFG, middle frontal gyrus; IFG, inferior frontal gyrus; PrG, precentral gyrus; MTG, middle temporal gyrus; ITG, inferior temporal gyrus; IPL, inferior parietal lobule; CG, cingulate gyrus.

#### FEDN MDD patients versus healthy controls

For the FEDN patients with MDD, no significant global topological alterations were identified. We found that FEDN patients showed altered regional centrality in the bilateral thalamic subfields, compared with healthy controls (Fig. 1 and Table 2).

### Recurrent MDD patients versus healthy controls

There were no significant global topological differences between recurrent patients and healthy controls. Regarding the regional centralities, recurrent patients with MDD exhibited increased regional centralities in the right inferior frontal gyrus (IFG), left middle frontal gyrus (MFG), left PCG and left IPL, while decreased regional centralities were observed in the left medial SFG (Fig. 1 and Table 2).

### Network connectivity abnormalities

#### MDD patients versus healthy controls

**Fig. 2:**
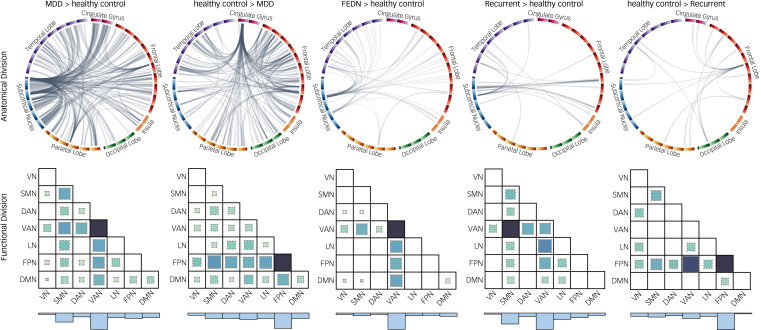
Abnormal connectivity patterns in MDD and relevant clinical subgroups. Each column corresponds to a group comparison. The upper panels show the significant focal connected network component and the anatomical divisions of the nodes. The lower panels show the divisions of the functional network of their nodes. The darker colour and the larger square denote the higher functional network connection weights, which were calculated as the ratio of the actual number of connections to the maximum number of connections. The histograms at the bottom show the sum of the network connection weights. MDD, major depressive disorder; FEDN, first-episode drug naïve; DMN, default mode network; FPN, frontoparietal network; VAN, ventral attention network; DAN, dorsal attention network; SMN, sensorimotor network; LN, limbic network; VN, visual network.

Two abnormal subnetwork components were identified in MDD patients, compared with healthy controls. One subnetwork component consisted of 129 nodes and 295 increased connectivity (*P* = 0.0003), which was anatomically involved in subcortical structures and functionally related to VAN. Another significant subnetwork component included 105 nodes and 203 decreased connectivity (*P* = 0.0006), which was anatomically involved in cingulate gyrus and was functionally related to the FPN (Fig. 2).

#### FEDN MDD patients versus healthy controls

FEDN patients with MDD exhibited one abnormal subnetwork component, compared with healthy controls. This subnetwork component included 75 nodes and 100 increased connectivity (*P* = 0.0020), which was anatomically involved in subcortical nuclei and functionally related to the VAN. No significant subnetwork component with increased connectivity was found in FEDN patients, compared with healthy controls (Fig. 2).

#### Recurrent MDD patients versus healthy controls

A subnetwork component including 22 nodes and 24 increased connectivity was identified in recurrent patients, compared with healthy controls (*P* = 0.0075). This subnetwork component was anatomically involved in subcortical areas and functionally related to the VAN. Another significant subnetwork component consisted of 17 nodes and 16 decreased connectivity (*P* = 0.0126) that was anatomically involved in frontal lobe and functionally related to the FPN. (Fig. 2).

### Individual-level classification performance

#### MDD patients versus healthy controls

**Fig. 3:**
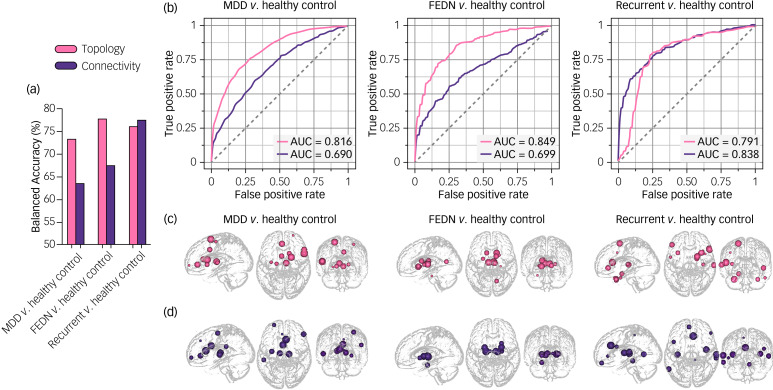
The individual-level classification performance of topology- and connectivity-based models. (a) comparison of balanced accuracy between the topology- and connectivity-based models across the different classification tasks; (b) receiver operating characteristic (ROC) curves of six models; (c) top ten brain regions that contributed most to the topology-based classification model; (d) top ten brain regions that contributed most to the connectivity-based classification model. The larger node corresponds to higher contribution. MDD, major depressive disorder; FEDN, first-episode drug naïve; AUC, area under the ROC curve.

Based on network topological metrics, the SVM model achieved an accuracy of 73.3% (95%CI: 71.4–75.3%, AUC: 0.816) between MDD patients and healthy controls. When using network connectivity as features, the classification accuracy between MDD patients and healthy controls was 63.4% (95%CI: 61.3–65.6%, AUC: 0.690). The most salient regions for both models were consistently located in the frontal gyrus, cingulate gyrus, PCG and thalamus (Fig. 3 and Supplementary Table 4).

#### FEDN MDD patients versus healthy controls

Based on network topology, FEDN patients were distinguished from healthy controls with an accuracy of 77.7% (95% CI: 74.8–80.6%, AUC: 0.849). With regard to network connectivity features, we observed a classification accuracy of 67.5% (95%CI: 64.2–70.7%, AUC: 0.677) between FEDN patients and healthy controls. The most salient regions were found in the subcortical nuclei including thalamus and basal ganglia (Fig. 3 and Supplementary Table 5).

#### Recurrent MDD patients versus healthy controls

By setting network topological metrics as features, recurrent patients were differentiated from healthy controls with an accuracy of 76.1% (95% CI: 73.1–79.1%, AUC: 0.791). For the network connectivity features, the classification accuracy between recurrent patients and healthy controls was 77.53% (95%CI: 74.6–80.5%, AUC: 0.838). The regions contributing most to classification between recurrent patients and healthy controls were distributed in the frontal, temporal and limbic regions (Fig. 3 and Supplementary Table 6).

### Associations with symptom severity

**Fig. 4:**
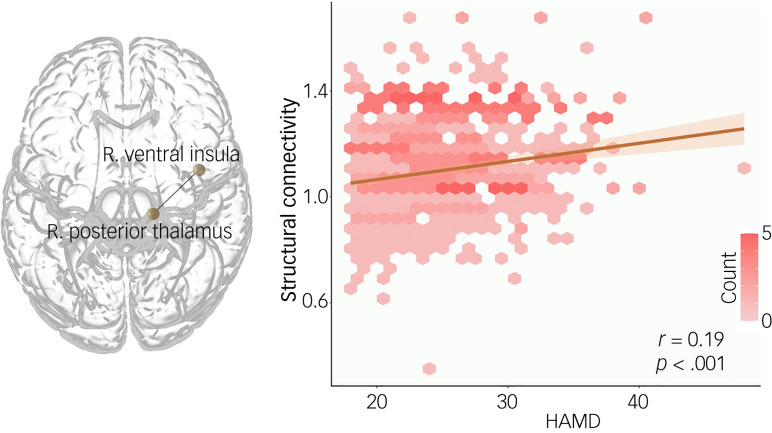
Correlation between the depressive symptom severity and thalamus-insula structural connectivity. The anatomical location of the connectivity with significant associations is shown in the left panel, and the scatterplot coloured by density shows the positive associations in the right panel. R, right; HAMD, Hamilton depression rating scale.

Structural connectivity between the posterior parietal thalamus and the ventral granular insula was positively associated with HAMD-17 total score (*N* = 822, *r* = 0.169, corrected *P* = 0.035). We did not detect significant correlations between topological metrics and depressive symptom severity (Fig. 4).

### Reproducibility results

Applying different analytic strategies to control potentially confounding factors did not change our main findings. The results of the reproducibility tests are shown in Supplementary Figure 3 and Tables 7–10.

## Discussion

Based on a large sample across 23 nationwide sites, this study, for the first time, investigated topological and connectional profiles of brain grey matter connectome and examined their diagnostic values in MDD and relevant clinical subtypes. For the network topological characteristics, MDD patients showed increased global efficiency and regional centralities of the temporal, parietal and thalamic areas, as well as decreased centralities of the medial prefrontal, anterior cingulate and precentral cortices compared to healthy controls. When looking at focal connections, we found increased connectivity within the cortical-subcortical circuit and decreased connectivity between the frontal and cingulate areas. Regarding the FEDN-MDD subgroup, both topological and connectional abnormalities were restricted to the subcortical structures, especially the thalamus, while disruption of iSCN in the recurrent MDD patients was primarily distributed in the frontal and parietal regions. Topological features showed potential for individual-level characterisation of MDD, including FEDN and recurrent patients, with classification accuracies around 75%. Connectional features of iSCN could achieve comparable accuracy for the recurrent MDD patients, but poorer performance was observed for the entire MDD and FEDN-MDD groups.

One key challenge in interpreting brain grey matter connectome is the lack of clarity of the neurobiological underpinnings of structural covariance based on imaging. The basic point is that brain structural covariance patterns are shaped during early childhood and inherently reflect neurodevelopmental coordination and maturational synchronisation between regions.^24^ A bundle of factors can alter the iSCNs across the lifespan, such as genetics, cognition, behaviour and plasticity,^24^ of which all have been involved in the pathogenesis of MDD. In addition, numerous studies have demonstrated that functional connectivity measuring interregional synchronisation of neuronal activities as well as anatomical connectivity representing white matter tracts is associated with the pathophysiology of MDD.^25^ Indeed, iSCNs can also be influenced by this brain connectivity and partially recapitulate functional and white matter networks, as such wiring can induce synaptogenesis, and synapses have mutually trophic and protective effects on connected neurons.^26,27^ Thus, examining the organisation and architecture of structural covariance patterns in MDD could provide a more comprehensive insight into the neural mechanisms, adding to our connectome-level understanding of the disease.

Brain grey matter connectome typically conforms to the small-world architecture that underlies an optimal organisation of global integration and local segregation. Compared with healthy controls, MDD patients exhibited increased global efficiency, which indicated higher network integration and consequently disrupted the balance between integration and segregation.^28^ One review of brain connectome studies summarised four canonical network configuration modes comprising regularisation, randomisation, and weaker and stronger small-worldisation.^29^ Enhanced integration and preserved segregation represent a shift towards the randomisation pattern of grey matter connectome in MDD,^29^ which has been consistently observed for functional networks in MDD.^30^ The randomisation may suggest that more resources are allocated for global information processing, and local specialised communication is limited, leading to impaired capability of fault tolerance.^31^

Altered regional centralities of the PCG and MCC were identified in MDD patients compared with healthy controls. Both multisite investigations and large-scale meta-analyses have supported the relationship between depression and structural abnormalities in these areas.^5,32^ The PCG, as part of the primary motor cortex, has been suggested to be involved in psychomotor retardation in MDD. It is notable that structural alterations in the PCG were identified in both currently depressed and remitted patients, representing a state-independent imaging biomarker of MDD.^33^ The MCC (especially the anterior part) plays an important role in psychological processes related to MDD, such as negative emotion, cognitive control, social information processing and reward-related decision-making. One previous study found significant correlation between reduction in depression severity and changes in the degree centrality of the MCC following 6 weeks of psychotherapy.^34^ Another study reported that neural activation and eigenvector centrality of the MCC showed predictive capability for remission to antidepressants in late-life depression.^35^ These findings suggest the MCC may be a promising region for monitoring and predicting treatment response.

In addition, abnormal topological metrics of the thalamus and DMN regions (i.e. mPFC, IPL and LTC) were also associated with MDD and distributed in different clinical subgroups. Specifically, FEDN patients showed aberrant centralities in the thalamus, while DMN abnormalities were found in recurrent patients. The thalamus is a key region regulating memory, emotion and arousal. One study found that SCN properties of the thalamus can serve as a trait-like signature of FEDN MDD, irrespective of the current state (depressed or remitted).^11^ Notably, altered iSCN centralities were identified only in several thalamic subfields. Another multisite study consistently found increased GMV in a few thalamic subfields but not in the entire thalamus.^36^ Heterogenous changes across the thalamic nuclei might extend our understanding of the neural basis of depression. In line with our findings, two multisite MRI studies suggested that DMN dysfunction in the entire MDD population was primarily driven by recurrent patients.^37,38^ The DMN has received the most attention in the context of MDD neurobiology because of its linkage to rumination which is the core phenomenology of depressive behaviours. Previous studies have demonstrated that antidepressants can modulate and change the DMN connectivity,^39^ suggesting that specific MDD findings in recurrent patients might result from longer-term treatment exposure.

For the network connectional profiles, we found increased connectivity within VAN regions and decreased connectivity related to FPN regions. Convergent patterns of increased connectivity related to VAN regions were identified in both FEDN and recurrent patients, while decreased connectivity related to FPN regions was only observed in recurrent MDD. The VAN is also known as the salience network involved in attention towards salient events. Overactivated VAN can lead to difficulties in the adaptive regulation of negative emotional events. Since mood symptoms are the leading feature of MDD which could be present at any illness stage, both FEDN and recurrent patients showed hyperconnectivity of the VAN. The FPN is a network related to top-down cognitive control. A large-scale meta-analysis found that deficits in cognitive performance persist and worsen with repeated depressive episodes.^40^ This may partially explain why significant FPN abnormalities were exclusively observed only in recurrent MDD.

The individualised classification implemented by machine learning revealed that the topological metrics successfully distinguished between patients and controls with excellent accuracy of over 70% in all groups and subgroups, which could serve as more reliable features in MDD diagnosis. Notably, although no significant global topological abnormalities were observed for the FEDN and recurrent subgroups, our topology-based model demonstrates the ability to characterise both FEDN and recurrent patients with remarkable accuracies exceeding 75%. It is noteworthy that all pivotal features contributing to this classification are regional topological centralities, suggesting that the classification procedure is predominantly influenced by nodal metrics. Global topology may exert minimal impact on the model performance in our analyses. For the clinical associations, no topological metrics were significantly associated with depressive symptom ratings. The connectivity between the thalamus and insula can serve as a promising indicator of symptom severity. Topological features represent higher-level architecture of iSCN which enables the tolerance of individual differences, while connectional features are a lower-level component of iSCN which may be sensitive to individual differences.

### Limitations

The current study has a few limitations. First, this is a cross-sectional and retrospective study that is limited to the investigation of illness progression models and neuropharmacological mechanisms in MDD. Future prospective and longitudinal neuroimaging studies with a large sample size are warranted. Second, our current findings are based on a data-set from Chinese participants. Relevant interpretations and conclusions applied to other ethnic/racial groups should be cautious. Future studies on Western depression patients could reveal commonalities and differences. Third, grey matter connectome was constructed based on GMV in our study. Structural covariance patterns estimated using other brain morphometric metrics, such as cortical thickness and surface may provide different biological underpinnings of MDD. Fourth, the episode status and medication history of most MDD patients were unclear, potentially influencing our main findings. Although we conducted subgroup analysis on FEDN MDD patients, the substantially reduced sample size compared to the overall data-set limits the robustness of our findings. Future neuroimaging studies focusing on a large and well-defined cohort of FEDN patients are essential to yield more robust and less biased insights into the neural mechanisms of MDD.

### Implications

Based on a sufficiently powered multisite data-set, we comprehensively investigated abnormalities of the grey matter connectome in MDD. Network topological deficits and focal connectional abnormalities exhibited unique patterns in FEDN and recurrent patients with MDD. Topological features enabled more reliable performance in illness diagnosis. Specific structural connection could serve as an indicator of illness severity. Our findings advance the current understanding of the connectome-level neurobiological mechanisms of MDD, providing a solid basis for future development of diagnosis and therapeutic targets.

## Supporting information

Long et al. supplementary material Long et al. supplementary material

## Data Availability

The data-set used in this study is openly available from the REST-meta-MDD consortium (http://rfmri.org/REST-meta-MDD).
