# Supplementary material for: Impaired topology and connectivity of grey matter structural networks in major depressive disorder: evidence from a multi-site neuroimaging data-set
Source: Br J Psychiatry. 2024 May;224(5):170–8. doi: 10.1192/bjp.2024.41 (PMC11039554; doi:10.1192/bjp.2024.41)
Supplement: Long et al. supplementary material [file S0007125024000412sup001.docx]

**Online Supplementary Materials**

**Table of contents**

[Supplementary Methods 2](#_Toc155951636)

[I. Data Selection 2](#_Toc155951637)

[II. Network Topological Metrics 2](#_Toc155951638)

[III. ComBat Harmonization 4](#_Toc155951639)

[IV. Support Vector Machine 4](#_Toc155951640)

[V. Reproducibility Tests 5](#_Toc155951641)

[Supplementary Table 1 6](#_Toc155951642)

[Supplementary Table 4 9](#_Toc155951643)

[Supplementary Table 5 10](#_Toc155951644)

[Supplementary Table 6 11](#_Toc155951645)

[Supplementary Table 7 12](#_Toc155951646)

[Supplementary Table 8 13](#_Toc155951647)

[Supplementary Table 9 14](#_Toc155951648)

[Supplementary Table 10 15](#_Toc155951649)

[Supplementary Figure 1 16](#_Toc155951650)

[Supplementary Figure 2 17](#_Toc155951651)

[Supplementary Figure 3 18](#_Toc155951652)

[References 19](#_Toc155951653)

# Supplementary Methods

## I. Data Selection

To control the data quality and avoid introduction of bias, subjects were selected from a total of 1300 MDD patients and 1128 healthy controls (HC) as follows: (1) subjects older than 65 years or younger than 18 years were excluded; (2) subjects with missing information on age sex were excluded; (3) low quality images detected by visual inspection were discarded; (4) one duplicated site (detected after consortium data sharing) was discarded; (5) subjects with HAMD-17 score less than 18.

## II. Network Topological Metrics

1. Global efficiency (*E*) is a measure of network integration which evaluates the efficiency of whole-network information exchange:

$$E=\frac{1}{N}\sum_{i\in G} E\left( i \right)= \frac{1}{N}\sum_{i\in G} \frac{\sum_{j\in G,j\neq i} d_{ij}^{-1}}{N-1}$$

where *E(i)* is the efficiency of node *i*, and *d_ij_* is the shortest weighted path between node *i* and *j*.

1. Clustering coefficient (*C*) is a measure of network segregation which measures the possibility of one node’s neighbors that are also mutually connected:

$$C=\frac{1}{N}\sum_{i\in G} C(i)$$

$$C(i)=\frac{1}{N}\sum_{i\in G} \frac{2t_{i}}{k_{i}(k_{i}-1)}$$

where *C(i)* is the clustering coefficient of node *i*, *K_i_* is the degree of node *i*, and *t*_i_ is the number of triangles around node *i*.

1. Small-worldness (*S*) is a measure of network small-worldness which assesses the balance between global integration and local segregation:

$$S=\frac{C_{real}/C_{rand}}{L_{real}/L_{rand}}$$

where *C_real_* and *L_real_* are the clustering coefficient and characteristic path length of the tested network, and *C_rand_* and *L_rand_* are the clustering coefficient and characteristic path length of the simulated random network. Small-world networks often have *S* ≫ 1.

1. Modularity (*Q*) is a measure of network modularity which reflects the degree to which the network may be subdivided into such clearly delineated and nonoverlapping groups:

$$Q=\sum_{u\in M} [e_{uu}-(\sum_{v\in M} e_{uv})]$$

where the network is fully subdivided into a set of nonoverlapping modules *M*, e_uu_ is the proportion of all links that connect nodes within module *u,* and *e_uv_* is the proportion of all links that connect nodes in module *u* with nodes in module *v*.

1. Assortativity (*r*) is a measure of network resilience which quantifies the tendency of nodes being connected to nodes with similar degree in a complex network:

$$r=\frac{l^{-1}\sum_{(i,j)\in L} k_{i}k_{j}-{[l^{-1}\sum_{\left( i,j \right)\in L} \frac{1}{2}(k_{i}+k_{j})]}^{2}}{l^{-1}\sum_{(i,j)\in L} \frac{1}{2}(k_{i}^{2}+k_{j}^{2})-{[l^{-1}\sum_{\left( i,j \right)\in L} \frac{1}{2}(k_{i}+k_{j})]}^{2}}$$

where *l* is the total number of connections, *L* is the connection that connects nodes *i* and *j*, and *k_i_* is the degree of node *i.*

1. Regional centralities including degree centrality (*DC*), betweenness centrality (*BC*) and eigenvector centrality (*EC*) variously assess importance of individual nodes. *DC* measures the connection of the index node with all the other nodes in a graph. *BC* is determined to assess the influence of a given node on information flow in the graph. *EC* is a more sophisticated index that assesses the sum of the centralities of its direct neighbors.

$$DC=\sum_{i\in G,i\neq j} a_{ij}$$

where a_ij_ denotes the connection status (0 or 1) between node *i* and *j*.

$$BC=\frac{1}{(N-1)(N-2)}\sum_{i\neq j\neq m\in G} \frac{\theta_{jm}(i)}{\theta_{jm}}$$

where θ_jm_ is the total number of shortest paths between node *j* and *m*, and θ_jm_(i) is the number of those shortest paths that path through node *i*.

$$EC= \frac{1}{\lambda_{1}}A\mu_{1}= \frac{1}{\lambda_{1}}\sum_{j=1}^{N} a_{ij}\mu_{1}(j)$$

Where *μ_1_(j)* is the *j*-th component of the first eigenvector of the adjacency matrix *A*, and *λ_1_* corresponds to the first eigenvalue (called the principal eigenvalue).

## III. ComBat Harmonization

In general, the site effects derived from varied scanners and sequence parameters may add noise to the image data and interfere with the statistical analysis. To remove this unwanted site effect and expose the actual abnormalities, we used a known harmonization method called ComBat. ComBat was originally developed to adjust batch effects in genomic studies^1^. Since then, it has been validated as an effective method for reducing site-related variability in multi-site structural, functional and diffusion MRI data^2-4^. ComBat is essentially a multivariate linear mixed effects regression model with additive and multiplicative terms for site effect. Empirical Bayes was used during the modelling process to improve the estimation of biological and site effect parameters. By removing site-effect variance and preserving biological variance of interest, ComBat provides a balanced way simultaneously to correct measurements from multi-site data and avoid overcorrection on important biological variance. Herein, we performed the ComBat harmonization on each topological and connectivity value. Age, sex, group were included as covariates of interest to be protected. For each measure, the ComBat model can be written as:

$$y_{ij}= \alpha+ X_{ij}^{T}\beta+ \gamma_{i}+ \delta_{i}\epsilon_{ij}$$

where y*_ij_* represents the measure in stie *i*, participant *j*, α denotes average measure value, X*_ij_^T^* is a design matrix for the variables of interest (e.g., age, sex, and group), and β is a vector of regression coefficients corresponding to X. In general, we assume that the residual term ϵ*_ij_* have mean 0. The terms γ*_i_* and δ*_i_* represent the additive (or location parameter) and multiplicative (or scale parameter) site effects of site *i*, respectively. The ComBat‐harmonized connectivity value was then determined as:

$$y_{ij}^{combat}= \frac{y_{ij- \hat{\alpha}-X_{ij}\hat{\beta}-\gamma_{i}^{*}}}{\delta_{i}^{*}}+\hat{\alpha}+X_{ij}\hat{\beta}$$

The site effects terms γ_i_^*^ and δ_i_^*^ are estimated by using empirical Bayes. ComBat simultaneously

models and estimates biological and nonbiological terms and algebraically removes the estimated

additive and multiplicative site effects.

## IV. Support Vector Machine

We separately used harmonized network topological metrics and connectivity values to train the classifiers based on support vector machine (SVM). The SVM model was implemented using the Python library scikit-learn (version 0.24.2). We chose to use SVM because it is the most common machine learning algorithm used in the neuroimaging community^5^. By finding the hyperplane maximizing the margin between binary classes in the feature space, SVM can learn the classification strategy from the training set, be optimized and fine-tuned with the validation set, and make individual classification decisions in a test set. The topological feature set contained 5 global metrics and three regional centralities of 246 regions, resulting in a total of n=743 features. The connectivity feature set contained a total of n=30135 features (i.e., 246 × (246 - 1)/2). Since the large amount of connectivity features might lead to overfitting, we applied feature selection to focus on connectivity showing significant between-group differences (P < 0.001). Ten-fold stratified cross-validation was applied to split the training and test sets. In this method, the participants were divided into 10 non-overlapping partitions, each with the same proportion of each class. In each one of the ten iterations of the cross-validation, nine partitions were used as the training set to train the SVM, and then the trained model was used to obtain predictions in the remaining one partition. A linear kernel was used to avoid the risk of overfitting. The hyperparameter C was determined via grid search on a set of values (i.e., [10^-3^, 10^-2^, 10^-1^, 1, 10^1^, 10^2^, 10^3^]), and the grid search was performed using another nested 10-fold stratified cross-validation within the training set. Classification performance was examined based on accuracy, sensitivity, specificity and AUC across 10 folds. The accuracy was determined as the percentage of correctly classified individuals among all study subjects. The sensitivity and specificity were used to indicate the percentage of correct classifications in positive and negative classes, respectively. For the estimation of AUC, we plotted receiver operating characteristic (ROC) curve showing the classification performance at all classification thresholds according to true positive rate (i.e., sensitivity) and false positive rate (i.e., 1 – specificity). The AUC value was thus calculated as the area under ROC curve to provide an aggregate measure of performance irrespective classification thresholds selection.

Supplementary Table 1 Scanner and acquisition parameters for each site.

| Site | Scanner | Field of view | TR  (ms) | TE  (ms) | Flip angle  (°) | Thickness  (mm) | Gap  (mm) | No. of axial slices | No. of Volumes | Voxel size  (mm^3^) |
| --- | --- | --- | --- | --- | --- | --- | --- | --- | --- | --- |
| 1 | Siemens Tim Trio (3T) | 210×210 | 2000 | 30 | 90 | 4 | 0.8 | 30 | 210 | 3.28×3.28×4.8 |
| 2 | Philips Achieva (3T) | 240×240 | 2000 | 30 | 90 | 4 | - | 37 | 200 | 1.67×1.67×4 |
| 3 | Siemens Magnetom Symphony (1.5 T) | 240×240 | 2000 | 40 | 90 | 5 | 1.25 | 26 | 150 | 3.75×3.75×6.25 |
| 4 | GE Signa (3T) | 240×240 | 3000 | 30 | 90 | 5 | - | 22 | 100 | 3.75×3.75×5 |
| 5 | Siemens Tim Trio (3T) | 230×230 | 2000 | 30 | 70 | 4 | - | 33 | 180 | 3.59×3.59×4 |
| 6 | GE Discovery MR750 (3T) | 220×220 | 2000 | 30 | 90 | 3.2 | - | 37 | 184 | 2.29×2.29×3.2 |
| 7 | GE Signa (3T) | 240×240 | 2000 | 30 | 90 | 3 | - | 35 | 200 | 3.75×3.75×3 |
| 8 | GE Discovery MR750 (3T) | 240×240 | 2000 | 25 | 90 | 3 | 1 | 35 | 200 | 3.75×3.75×4 |
| 9 | Siemens Tim Trio (3T) | 240×240 | 2000 | 30 | 90 | 3 | 1.52 | 32 | 212 | 3.75×3.75×4.52 |
| 10 | GE Signa (3T) | 240×240 | 2000 | 30 | 90 | 5 | - | 33 | 200 | 3.75×3.75×5 |
| 11 | GE Signa (3T) | 240×240 | 2000 | 30 | 90 | 5 | - | 33 | 240 | 3.75×3.75×4 |
| 12 | GE Excite (1.5T) | 256×256 | 2500 | 35 | 90 | 4 | - | 36 | 150 | 4×4×4 |
| 13 | Siemens Tim Trio (3T) | 240×240 | 2500 | 25 | 90 | 3.5 | - | 39 | 200 | 3.75×3.75×3.5 |
| 14 | Siemens Verio (3T) | 240×240 | 2000 | 25 | 90 | 4 | - | 36 | 240 | 3.75×3.75×4 |
| 15 | GE Signa (3T) | 240×240 | 2000 | 30 | 90 | 5 | - | 30 | 200 | 3.75×3.75×5 |
| 16 | GE Signa (3T) | 240×240 | 2000 | 40 | 90 | 4 | - | 33 | 240 | 3.75×3.75×4 |
| 17 | Philips Achieva (3T) | 240×240 | 2000 | 35 | 90 | 5 | 1 | 24 | 200 | 1.67×1.67×6 |
| 18 | GE Signa (3T) | 220×220 | 2000 | 22.5 | 30 | 4 | 0.6 | 33 | 240 | 3.44×3.44×4.6 |
| 19 | Siemens Tim Trio (3T) | 220×220 | 2000 | 30 | 90 | 3 | 1 | 32 | 242 | 3.44×3.44×4 |
| 20 | Siemens Tim Trio (3T) | 200×200 | 2000 | 30 | 90 | 3.5 | 0.7 | 33 | 240 | 3.12×3.12×4.2 |
| 21 | Philips Gyroscan Achieva (3T) | 240×240 | 2000 | 30 | 90 | 4 | - | 36 | 250 | 1.67×1.67×4 |
| 22 | Philips Achieva (3T) | 240×240 | 2000 | 30 | 90 | 4 | - | 38 | 240 | 3.75×3.75×4 |
| 23 | GE Signa (1.5T) | 240×240 | 2000 | 40 | 90 | 5 | 1 | 24 | 160 | 3.75×3.75×6 |

***Abbreviations:*** TR, repetition time; TE, echo time.

**Supplementary Table 2** Demographic and Clinical Characteristics of Included Sample in Subgroup Analysis of FEDN MDD Patients.

| Variable | FEDN MDD | HC | *p*-value |
| --- | --- | --- | --- |
| Sample Size (N) | 263 | 532 | - |
| Age (year, mean±SD) | 33.8±10.6 | 35.8±13.8 | .042 |
| Sex (N female, %) | 170 (64.6%) | 319 (60.0%) | .202 |
| HAMD (mean±SD) | 23.7±4.6 | - | - |
| Duration of Illness (month, mean±SD) | 16.2±30.0 | - | - |

***Abbreviations****:* FEDN, first-episode drug-naïve; MDD, major depressive disorder; HC, healthy controls; HAMD, Hamilton Depression Rating Scale.

**Supplementary Table 3** Demographic and Clinical Characteristics of Included Sample in Subgroup Analysis of Recurrent MDD.

| Variable | Recurrent MDD | HC | *p*-value |
| --- | --- | --- | --- |
| Sample Size (N) | 201 | 562 | - |
| Age (year, mean±SD) | 36.2±12.4 | 36.8±14.0 | .617 |
| Sex (N female, %) | 122 (60.7%) | 316 (56.2%) | .749 |
| HAMD (mean±SD) | 23.4±4.9 | - | - |
| Duration of Illness (month, mean±SD) | 83.3±79.5 | - | - |

***Abbreviations****:* MDD, major depressive disorder; HC, healthy controls; HAMD, Hamilton Depression Rating Scale.

| Rank | Regions | |
| --- | --- | --- |
|  | Topology-based model | Connectivity-based model |
| 1 | R IFG, ventral area 44 | R CG, rostroventral area 24 |
| 2 | R SFG, dorsolateral area 6 | L CG, rostroventral area 24 |
| 3 | R Medial prefrontal thalamus | R Lateral prefrontal thalamus |
| 4 | L CG, subgenual area 32 | L Lateral prefrontal thalamus |
| 5 | R IFG, caudal area 45 | R IFG, rostral area 45 |
| 6 | L CG, caudodorsal area 24 | L PrG, caudal dorsolateral area 6 |
| 7 | R Ventral caudate | L IPL, rostroventral area 40 |
| 8 | L Pre-motor thalamus | L MFG, lateral area 10 |
| 9 | L PrG, caudal dorsolateral area 6 | L Rostral temporal thalamus |
| 10 | R MFG, lateral area 10 | L SFG, medial area 9 |

Supplementary Table 4 Top 10 regions for topology- and connectivity-based models distinguishing between MDD and HC.

***Abbreviations***: IFG, inferior frontal gyrus; MFG, middle frontal gyrus; SFG, superior frontal gyrus; CG, cingulate gyrus; PrG, Precentral gyrus; IPL, inferior parietal lobule.

| Rank | Regions | |
| --- | --- | --- |
|  | Topology-based model | Connectivity-based model |
| 1 | R Medial prefrontal thalamus | L Pre-motor thalamus |
| 2 | L Medial prefrontal thalamus | R Pre-motor thalamus |
| 3 | R Ventral caudate | R Globus pallidus |
| 4 | R Caudal temporal thalamus | L Globus pallidus |
| 5 | R Dorsal caudate | R Medial prefrontal thalamus |
| 6 | L Ventral caudate | R Rostral hippocampus |
| 7 | R MvOcC, ventromedial parietooccipital sulcus | R Ventromedial putamen |
| 8 | L Globus pallidus | R Rostral temporal thalamus |
| 9 | R Ventromedial putamen | L Medial prefrontal thalamus |
| 10 | R PhG, rostral area 35/36 | L Dorsolateral putamen |

Supplementary Table 5 Top 10 regions for topology- and connectivity-based models distinguishing between FEDN MDD and HC.

***Abbreviations***: MvOcC, Medioventral occipital cortex; PhG, Parahippocampal gyrus.

| Rank | Regions | |
| --- | --- | --- |
|  | Topology-based model | Connectivity-based model |
| 1 | R IFG, caudal area 45 | R CG, pregenual area 32 |
| 2 | R STG, medial area 38 | R STG, caudal area 22 |
| 3 | R SFG, dorsolateral area 6 | R Dorsal agranular insula |
| 4 | R STG, caudal area 22 | L Hypergranular insula |
| 5 | L IFG, rostral area 45 | L MTG, dorsolateral area 37 |
| 6 | R Medial amygdala | R MTG, caudal area 21 |
| 7 | L STG, medial area 38 | L OrG, medial area 11 |
| 8 | R Hypergranular insula | L IFG, dorsal area 44 |
| 9 | L MTG, rostral area 21 | R IFG, rostral area 45 |
| 10 | R MFG, inferior frontal junction | R PCL, area 1/2/3 (lower limb region) |

Supplementary Table 6 Top 10 regions for topology- and connectivity-based models distinguishing between recurrent MDD and HC.

***Abbreviations***: IFG, inferior frontal gyrus; MFG, middle frontal gyrus; STG, superior temporal gyrus; MTG, middle temporal gyrus; CG, cingulate gyrus; OrG, Orbital gyrus; PCL, paracentral lobule.

Supplementary Table 7 Reproducibility tests for regional topological abnormalities using AAL3 atlas.

| Regions | Metrics | Cohen's *d* | *p*-value  (Bonferroni corrected) |
| --- | --- | --- | --- |
| ***MDD < HC*** | | | |
| L medial SFG | Degree centrality | -0.18 | 0.0105 |
| L anterior OFC | Degree centrality | -0.16 | 0.0207 |
| R superior ACC | Eigenvector centrality | -0.25 | < 0.0001 |
| ***MDD > HC*** | | | |
| L IPL | Betweenness centrality | 0.20 | 0.0021 |
|  | Eigenvector centrality | 0.20 | 0.0016 |
| L MDI Thalamus | Degree centrality | 0.22 | < 0.0001 |
|  | Eigenvector centrality | 0.33 | < 0.0001 |
| R MTG | Eigenvector centrality | 0.17 | 0.0170 |
| ***FEDN < HC*** | | | |
| L Intralaminar Thalamus | Degree centrality | -0.32 | 0.0149 |
| L PuL Thalamus | Degree centrality | -0.32 | 0.0148 |
| L LGN Thalamus | Eigenvector centrality | -0.39 | 0.0001 |
| ***FEDN > HC*** |  |  |  |
| L MDI Thalamus | Eigenvector centrality | 0.34 | 0.0076 |
| ***Recurrent < HC*** |  |  |  |
| L medial SFG | Eigenvector centrality | -0.48 | < 0.0001 |
| ***Recurrent > HC*** |  |  |  |
| L orbital IFG | Eigenvector centrality | 0.32 | 0.0316 |
| L IPL | Eigenvector centrality | 0.31 | 0.0140 |

***Abbreviations***: AAL3, automated anatomical labelling atlas 3; MDD, major depressive disorder; HC, healthy controls; FEDN, first-episode drug-naïve; L, left; R, right; SFG, superior frontal gyrus; OFC, orbitofrontal cortex; ACC, anterior cingulate cortex; MDI, Mediodorsal lateral parvocellular; MTG, middle temporal gyrus; PuL, Pulvinar lateral Pulvinar; LGN, Lateral geniculate; IFG, inferior frontal gyrus; IPL, inferior parietal lobule.

Supplementary Table 8 Reproducibility tests for regional topological abnormalities using Brainnetome atlas including cerebellar regions.

| Regions | Metrics | Cohen's *d* | *p*-value  (Bonferroni corrected) |
| --- | --- | --- | --- |
| ***MDD < HC*** | | | |
| L SFG, medial area 8 | Degree centrality | -0.17 | 0.0231 |
| L IFG, opercular area 44 | Betweenness centrality | -0.18 | 0.0277 |
| L PrG, area 4 | Eigenvector centrality | -0.20 | 0.0031 |
| L CG, rostroventral area 24 | Degree centrality | -0.16 | 0.0492 |
|  | Eigenvector centrality | -0.19 | 0.0024 |
| R CG, rostroventral area 24 | Degree centrality | -0.19 | 0.0075 |
|  | Eigenvector centrality | -0.19 | 0.0019 |
| ***MDD > HC*** | | | |
| R MFG, area 46 | Degree centrality | 0.20 | 0.0021 |
| R MTG, caudal area 21 | Degree centrality | 0.20 | 0.0016 |
| R IPL, rostroventral area 40 | Degree centrality | 0.22 | < 0.0001 |
|  | Eigenvector centrality | 0.19 | 0.0154 |
| L medial prefrontal thalamus | Degree centrality | 0.17 | 0.0170 |
|  | Eigenvector centrality | 0.19 | 0.0047 |
| ***FEDN < HC*** | | | |
| R pre-motor thalamus | Eigenvector centrality | -0.37 | 0.0007 |
| R lateral prefrontal thalamus | Eigenvector centrality | -0.34 | 0.0003 |
| ***Recurrent < HC*** |  |  |  |
| L SFG, medial area 6 | Betweenness centrality | -0.33 | 0.0261 |
| ***Recurrent > HC*** |  |  |  |
| R IFG, rostral area 45 | Degree centrality | 0.33 | 0.0086 |
| L IPL, rostroventral area 40 | Degree centrality | 0.30 | 0.0406 |

***Abbreviations***: MDD, major depressive disorder; HC, healthy control; FEDN, first-episode drug-naïve; L, left; R, right; SFG, superior frontal gyrus; MFG, middle frontal gyrus; IFG, inferior frontal gyrus; PrG, precentral gyrus; MTG, middle temporal gyrus; IPL, inferior parietal lobule; CG, cingulate gyrus.

Supplementary Table 9 Reproducibility tests for regional topological abnormalities when using non-smoothed GMV maps for network construction.

| Regions | Metrics | Cohen's *d* | *p*-value  (Bonferroni corrected) |
| --- | --- | --- | --- |
| ***MDD < HC*** | | | |
| L SFG, medial area 8 | Degree centrality | -0.24 | < 0.0001 |
| L SFG, medial area 9 | Degree centrality | -0.19 | 0.0093 |
| L IFG, opercular area 44 | Betweenness centrality | -0.18 | 0.0352 |
| L PrG, caudal dorsolateral area 6 | Eigenvector centrality | -0.18 | 0.0089 |
| R STG, medial area 38 | Eigenvector centrality | -0.23 | 0.0005 |
| L STG, rostral area 22 | Eigenvector centrality | -0.17 | 0.0309 |
| L CG, rostroventral area 24 | Degree centrality | -0.20 | 0.0005 |
|  | Eigenvector centrality | -0.24 | < 0.0001 |
| R CG, rostroventral area 24 | Degree centrality | -0.21 | 0.0003 |
|  | Eigenvector centrality | -0.20 | 0.0005 |
| ***MDD > HC*** | | | |
| R MFG, dorsal area 9/46 | Degree centrality | 0.23 | < 0.0001 |
| L medial prefrontal thalamus | Degree centrality | 0.18 | 0.0327 |
| R MTG, caudal area 21 | Betweenness centrality | 0.18 | 0.0137 |
| L IPL, rostroventral area 40 | Eigenvector centrality | 0.17 | 0.0442 |
| R PoG, area 2 | Eigenvector centrality | 0.22 | 0.0002 |
| R BG, dorsolateral putamen | Eigenvector centrality | 0.18 | 0.0151 |
| ***FEDN < HC*** | | | |
| R lateral Prefrontal thalamus | Degree centrality | -0.35 | 0.0009 |
| ***FEDN > HC*** |  |  |  |
| R medial Prefrontal thalamus | Eigenvector centrality | 0.30 | 0.0480 |
| ***Recurrent < HC*** |  |  |  |
| L FuG, medioventral area 37 | Degree centrality | -0.32 | 0.0053 |
| L SFG, medial area 8 | Eigenvector centrality | -0.32 | 0.0055 |
| L PrG, caudal dorsolateral area 6 | Eigenvector centrality | -0.30 | 0.0157 |
| ***Recurrent > HC*** |  |  |  |
| R MFG, dorsal area 9/46 | Degree centrality | 0.36 | 0.0016 |
| R IFG, rostral area 45 | Degree centrality | 0.31 | 0.0481 |
| L PrG, area 4(upper limb region) | Eigenvector centrality | 0.29 | 0.0466 |
| L PCun, medial area 7 | Eigenvector centrality | 0.33 | 0.0083 |
| R PoG, area 2 | Eigenvector centrality | 0.41 | < 0.0001 |

***Abbreviations***: GMV, gray matter volume; MDD, major depressive disorder; HC, healthy control; FEDN, first-episode drug-naïve; SFG, superior frontal gyrus; MFG, middle frontal gyrus; IFG, inferior frontal gyrus; PrG, precentral gyrus; STF, superior temporal gyrus; MTG, middle temporal gyrus; IPL, inferior parietal lobule; CG, cingulate gyrus; BG, basal ganglia; PoG, postcentral gyrus; PCun, precuneus.

Supplementary Table 10 Reproducibility tests for regional topological abnormalities when adding total intracranial volume as covariate.

| Regions | Metrics | Cohen's *d* | *p*-value  (Bonferroni corrected) |
| --- | --- | --- | --- |
| ***MDD < HC*** | | | |
| L SFG, medial area 8 | Degree centrality | -0.19 | 0.0058 |
| L SFG, medial area 6 | Degree centrality | -0.17 | 0.0379 |
| L SFG, medial area 9 | Degree centrality | -0.18 | 0.0212 |
| L PrG, caudal dorsolateral area 6 | Degree centrality | -0.22 | 0.0004 |
|  | Eigenvector centrality | -0.20 | 0.0027 |
| L CG, rostroventral area 24 | Degree centrality | -0.25 | <0.0001 |
|  | Eigenvector centrality | -0.26 | <0.0001 |
| R CG, rostroventral area 24 | Degree centrality | -0.23 | <0.0001 |
|  | Eigenvector centrality | -0.23 | <0.0001 |
| ***MDD > HC*** | | | |
| R MTG, caudal area 21 | Degree centrality | 0.22 | 0.0006 |
|  | Betweenness centrality | 0.19 | 0.0155 |
| L ITG, extreme lateroventral area 37 | Degree centrality | 0.20 | 0.0039 |
|  | Eigenvector centrality | 0.19 | 0.0070 |
| L IPL, rostroventral area 40 | Degree centrality | 0.20 | 0.0026 |
| L medial prefrontal thalamus | Degree centrality | 0.21 | 0.0002 |
|  | Eigenvector centrality | 0.17 | 0.0125 |
| ***FEDN < HC*** | | | |
| R lateral prefrontal thalamus | Degree centrality | -0.33 | 0.0021 |
| R pre-motor thalamus | Eigenvector centrality | -0.35 | 0.0005 |
| ***FEDN > HC*** |  |  |  |
| L medial prefrontal thalamus | Betweenness centrality | 0.27 | 0.0298 |
| ***Recurrent < HC*** |  |  |  |
| L SFG, medial area 8 | Eigenvector centrality | -0.32 | 0.0127 |
| ***Recurrent > HC*** |  |  |  |
| L MFG, dorsal area 9/46 | Degree centrality | 0.33 | 0.0109 |
| R IFG, rostral area 45 | Degree centrality | 0.37 | 0.0012 |
|  | Eigenvector centrality | 0.33 | 0.0085 |
| L IPL, rostroventral area 40 | Degree centrality | 0.34 | 0.0058 |
| L PrG, upper limb area 4 | Eigenvector centrality | 0.32 | 0.0184 |

***Abbreviations***: MDD, major depressive disorder; HC, healthy control; FEDN, first-episode drug-naïve; L, left; R, right; SFG, superior frontal gyrus; MFG, middle frontal gyrus; IFG, inferior frontal gyrus; PrG, precentral gyrus; MTG, middle temporal gyrus; ITG, inferior temporal gyrus; IPL, inferior parietal lobule; CG, cingulate gyrus.


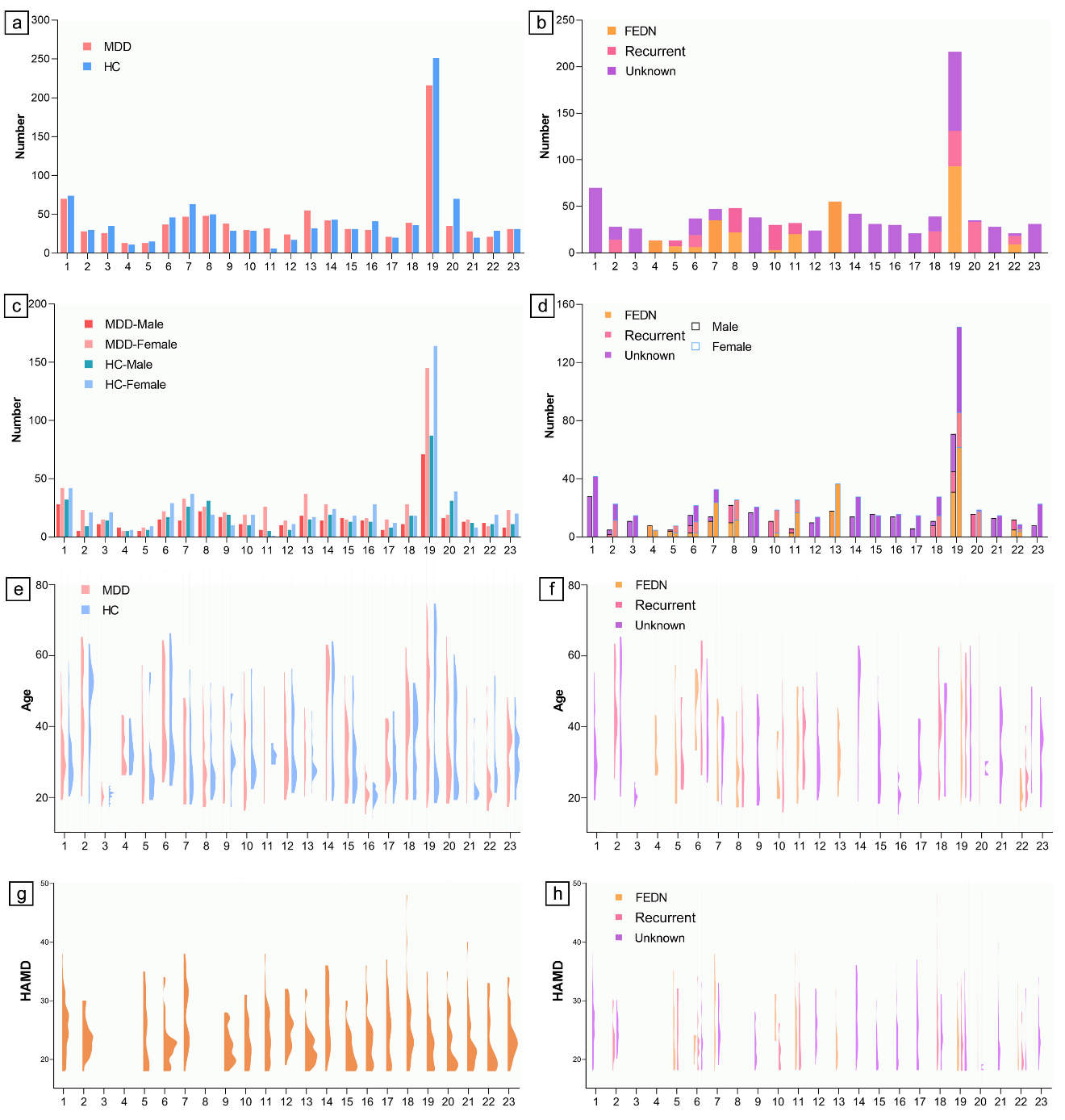
Supplementary Figure 1 Demographic and clinical characteristics at each site. A total of 1964 individuals were included from the multi-site dataset. Panels on the left side provide case-control information, and those on the right side provide information about MDD clinical subgroups. (a-b) sample size distribution; (c-d) sex distribution; (e-f) age distribution; (g-h) symptom severity assessed using HAMD-17.

***Abbreviations***: MDD, major depressive disorder; HC, healthy controls; FEDN, first-episode drug-naïve; HAMD, Hamilton Depression Rating Scale, 17 items.


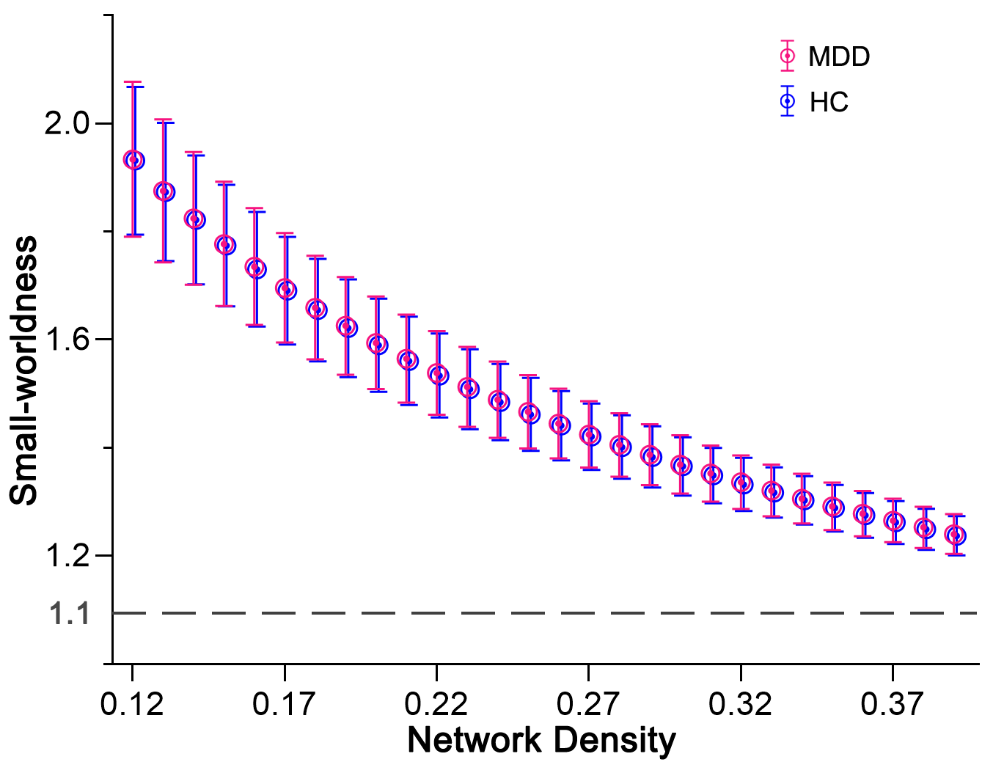


Supplementary Figure 2 Network small-worldness across the dynamic density thresholds. Each circle and error bar denote the mean and standard deviation of small-worldness at each density threshold, respectively.

***Abbreviations***: MDD, major depressive disorder; HC, healthy controls.


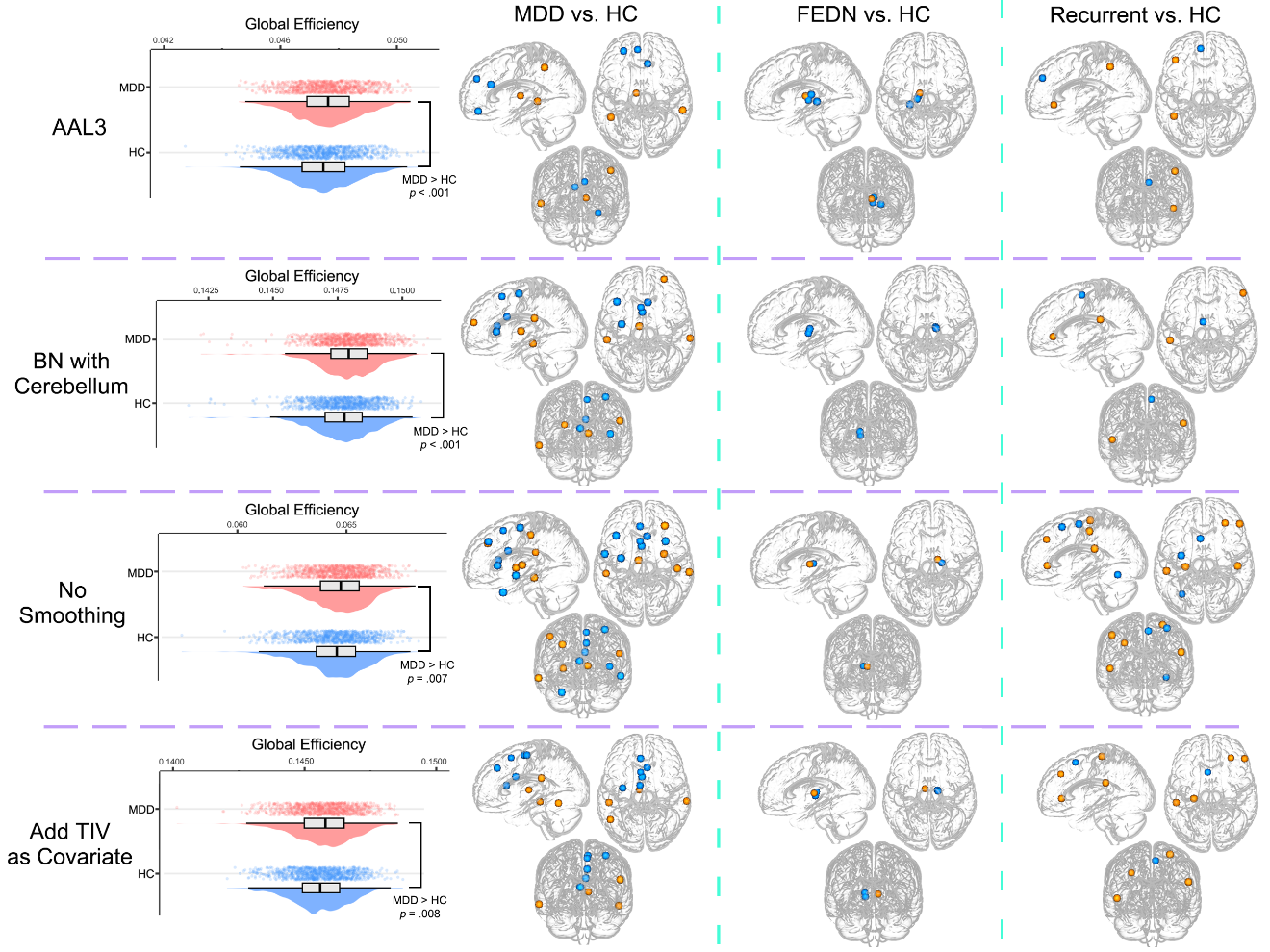
Supplementary Figure 3 Reproducibility tests for abnormal topological metrics. Significant between-group differences in topological metrics were examined when using the AAL3 atlas and Brainnetome atlas with cerebellum in the construction of iSCN, building up without spatial smoothing during image preprocessing, and adding TIV as additional covariate for statistical analysis. The orange nodes represent for regions that patients have significantly higher centralities than controls, and the blue ones represent the opposite.

***Abbreviations***: AAL3, automated anatomical labelling atlas 3; TIV, total intracranial volume; MDD, major depressive disorder; FEDN, first-episode drug naïve; HC, healthy controls; iSCN, individualized structural covariance network.

# References

1. Johnson, W.E., Li, C. & Rabinovic, A. Adjusting batch effects in microarray expression data using empirical Bayes methods. *Biostatistics (Oxford, England)* **8**, 118-127 (2007).

2. Yu, M.*, et al.* Statistical harmonization corrects site effects in functional connectivity measurements from multi-site fMRI data. *Human brain mapping* **39**, 4213-4227 (2018).

3. Fortin, J.P.*, et al.* Harmonization of cortical thickness measurements across scanners and sites. *NeuroImage* **167**, 104-120 (2018).

4. Fortin, J.P.*, et al.* Harmonization of multi-site diffusion tensor imaging data. *NeuroImage* **161**, 149-170 (2017).

5. Orrù, G., Pettersson-Yeo, W., Marquand, A.F., Sartori, G. & Mechelli, A. Using Support Vector Machine to identify imaging biomarkers of neurological and psychiatric disease: a critical review. *Neuroscience and biobehavioral reviews* **36**, 1140-1152 (2012).
